# First report of rare fungal keratitis: *Diaporthe aspalathi*

**DOI:** 10.1186/s12886-025-04538-2

**Published:** 2025-12-08

**Authors:** Dan Huang, Yanan Huo, Qi Dai, Xiaoyi Qin, Danyan Luo, Huiqin Wang

**Affiliations:** 1 Department of Ophthalmology, The Second Affiliated Hospital of Zhejiang University School of Medicine, Hangzhou, China; 2 Affiliated Eye Hospital of Wenzhou Medical College, Wenzhou, China; 3 Department of Ophthalmology, The First Affiliated Hospital of Zhejiang Chinese Medical University, Hangzhou, China; 4 The Second People’s Hospital of Quzhou, Quzhou, China

**Keywords:** *Diaporthe aspalathi*, Fungal keratitis, Penetrating keratoplasty

## Abstract

**Background:**

The plant fungal pathogen *Diaporthe aspalathi* is the causal agent of the southern stem canker disease in soybean. However, human infection caused by *D. aspalathi* has not been previously reported.

**Case presentation:**

We report a case of human keratitis caused by *D. aspalathi* on a 50-year-old woman. She presented to the hospital with redness, pain, decreased vision, and a focal white opacity in the left eye, which had been injured by a thorn 3.5 months earlier. Slit lamp examination revealed moderate conjunctival hyperemia, intact corneal epithelium, mild stromal edema, and thick white endothelial exudates in the inferior cornea. *D. aspalathi* was identified through DNA sequencing. The patient was treated with a combination of antifungal agents and therapeutic penetrating keratoplasty, resulting in scarring and vascularization of the graft.

**Conclusion:**

Our report shows a rare case of corneal infection caused by *D. aspalathi* and highlights the importance of early suspicion of fungal keratitis in patients with a history of corneal plant trauma.

## Introduction

*Diaporthe aspalathi*, a well-known plant fungus, is the causative agent of the southern stem canker disease in soybean (*Glycine max*) [1]; however, human infection caused by *D. aspalathi* has not been reported. Herein, we report a case of *D. aspalathi* infection in a patient presenting with fungal keratitis. We aim to alert clinicians and laboratorians to consider *D. aspalathi* as a potential cause of keratitis in humans. In particular, we provide data regarding the natural course, clinical manifestations, pathogen DNA sequence, and histopathological result for the diagnosis of the condition, as well as indicate potential modalities suitable for its treatment.

## Case description

A 50-year-old female presented with uncontrolled keratitis in her left eye, which had begun 3.5 months earlier after a thorn injury. Initial redness and discomfort persisted despite treatment with antibiotic eye drops. Two months post-injury, she developed pain, decreased vision, and a focal corneal opacity. A misdiagnosis of herpes simplex keratitis (HSK) led to a one-month course of oral acyclovir and multiple steroids (fluorometholone eye drops, 0.1%, topically; prednisolone acetate, 1%, topically; prednisone, orally; dexamethasone, intravenously) at other hospitals, which failed to resolve her symptoms and the lesion continued to enlarge.

**Fig. 1:**
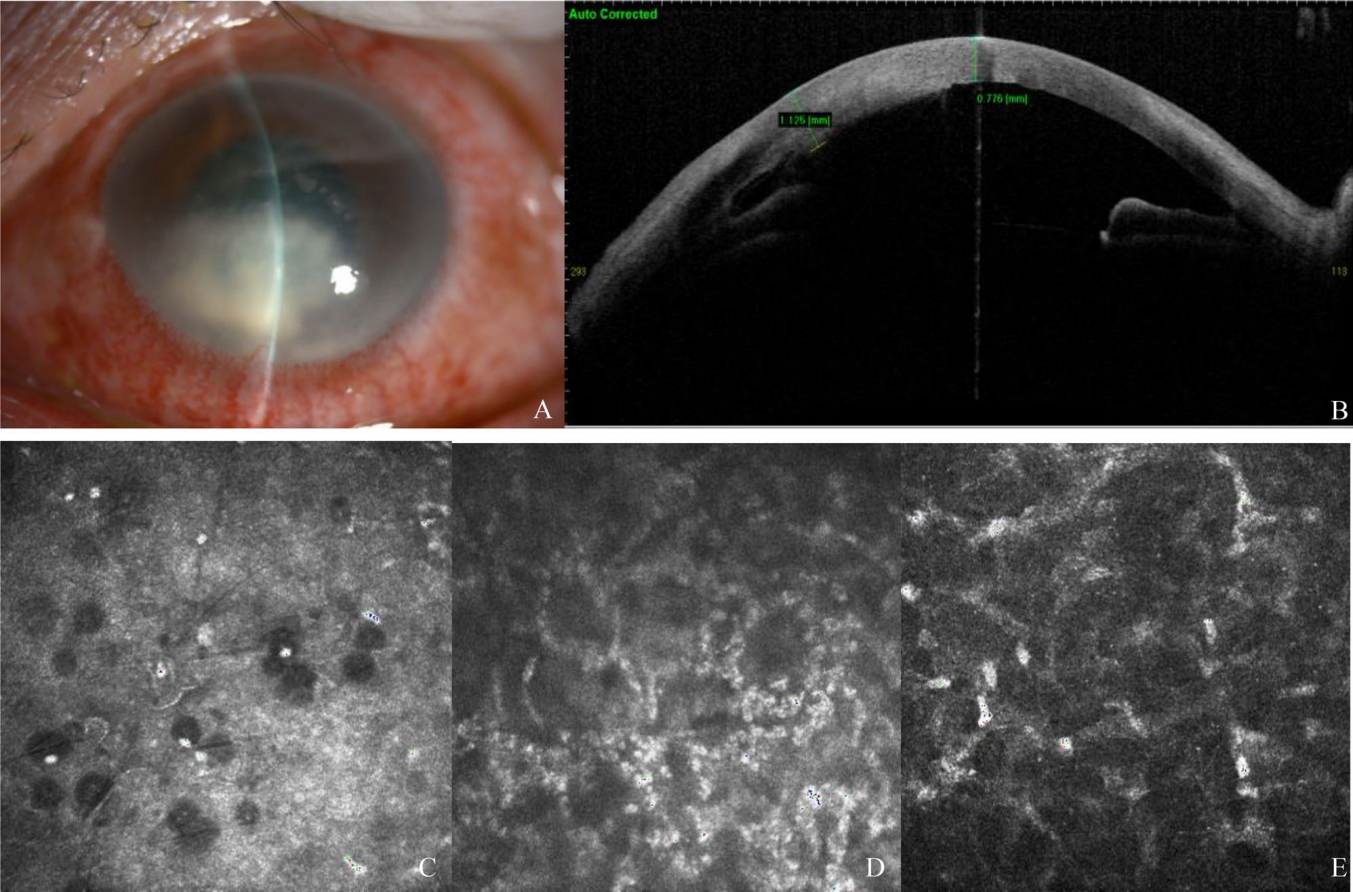
At the time of presentation **A**) Slit-lamp photographs (stromal edema and thick white endothelial exudates of the inferior cornea), **B**) Anterior segment optical coherence tomography (corneal edema and deep stromal infiltration), **C-E**) In vivo confocal microscopy negative for fungal hyphae **C(14** μm**)**:abnormal morphology of epithelial cells with dot-like and hyperreflective deposits, **D(237** μm**)**: infiltration of numerous inflammatory cells and partially activated stromal cells in the middle corneal stroma, **E(500** μm**)**:unclear endothelial cell layer

At presentation to our hospital, the patient’s visual acuity was hand motion in the left eye. Slit-lamp examination demonstrated moderate conjunctival hyperemia, intact corneal epithelium, mild stromal edema, and thick white endothelial exudates in the inferior cornea (Fig. 1A). The fundus was not visible due to corneal opacity. Anterior segment optical coherence tomography revealed corneal edema and deep stromal infiltration (Fig. 1B). In vivo confocal microscopy, which provided clear imaging to a depth of approximately 380-µm (with deeper structures becoming blurred), showed infiltration of numerous inflammatory cells in the mid-posterior stroma and an absence of hyphae (Fig. 1C-E). Fungal keratitis was strongly suspected in this case due to a slowly progressive corneal lesion following a thorn injury, a history of corticosteroid use, and a lack of response to antiviral therapy. As the lesion was in the deep cornea, corneal scraping was deemed unsuitable. Aqueous humor sample was extracted for metagenomic next-generation sequencing (mNGS), and all steroids were discontinued. The patient was treated with voriconazole (1%, topically; Vfend; Pfizer, New York, USA) hourly and itraconazole (200 mg, orally; Janssen, Xian, China) daily.

**Fig. 2:**
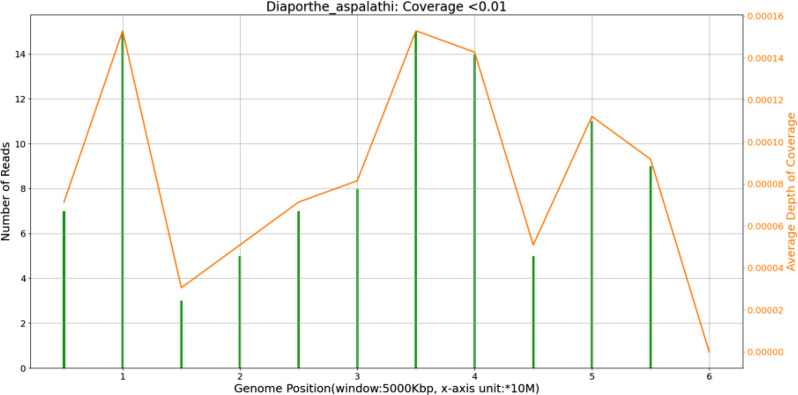
mNGS results. Specific sequences are evenly distributed on the genome, indicating pathogen detection authenticity and reliability

At 3 days after sample extraction, mNGS results revealed the presence of *D. aspalathi* (Fig. 2). The next day, the patient was administered voriconazole (1%, intrastromally and intracamerally). Over the next 7 days, corneal lesion size and endothelial exudate density increased. Ultrasound biomicroscopy revealed a foreign body in the inferior corneosclera. We removed the foreign body (i.e., the thorn) and administered voriconazole (1%, intrastromally and intracamerally) again. Thereafter, antifungal therapy with voriconazole (1%, topically) hourly and itraconazole (200 mg, orally) daily was continued.

**Fig. 3:**
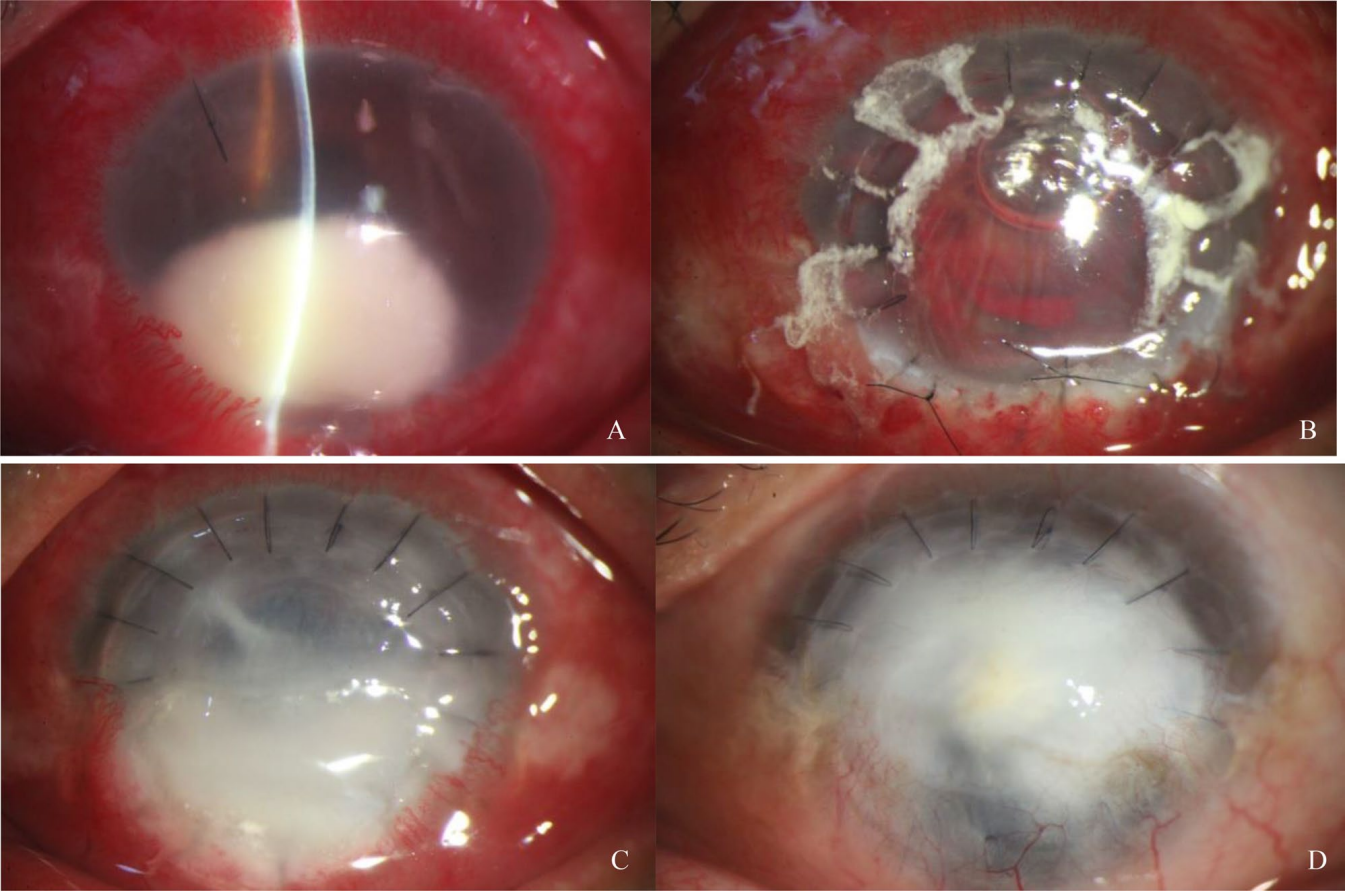
Slit-lamp photographs **A**) 1 day before surgery (worsening of corneal infection), **B**) 1 day after surgery (transparent graft with inferior corneoscleral tissue, anterior chamber hemorrhage, and white natamycin adhering to the corneal graft), **C**) 1 month after surgery (inferior graft with gradual infiltration, opacity, edema and stromal lysis), and **D**) 9 months after surgery (corneal scarring, vascularization, and inferior thinning)

Despite the treatment, the corneal infection continued to worsen (Fig. 3A). After 2 weeks of antifungal therapy, we performed therapeutic penetrating keratoplasty (Fig. 3B). After the washout of fibrinoid exudation and purulence from the anterior chamber with 2% voriconazole, the fibrinoid membranes on the surfaces of the crystalline lens and iris were peeled off. An 8-mm graft (containing a small amount of corneoscleral tissue) was sufficient to remove the focus of infection. During surgery, the excised host cornea was sent for microscopic examination, culture, and histopathological analysis. Direct microscopy with Giemsa stain revealed numerous hyphae. Treatment with voriconazole (200 mg, twice a day, orally; Vfend), voriconazole (2%, topically, every 30 min), and natamycin (5%, topically, every 30 min; Natacyn; Alcon Laboratories, Japan) was resumed postoperatively, along with tacrolimus eye drops (0.1%, four times daily; Talymus; Senju Pharmaceutical, Osaka, Japan). Corticosteroids were not administered postoperatively. At 1 week after surgery, the inferior graft demonstrated gradual infiltration, opacity, edema, and stromal lysis (Fig. 3C). Because of delayed reepithelialization, we performed amniotic membrane transplantation twice. Multiple confocal microscopy and corneal scraping examinations did not demonstrate hyphae. Antifungal therapy was continued for 3 months with gradual tapering until the epithelium was intact. At 3 months after surgery, treatment with topical steroid fluorometholone (0.1%; Santen Pharmaceutical) was initiated. At her recent follow-up visit (9 months after surgery), the cornea had developed scarring, vascularization, and inferior thinning (Fig. 3D) with visual acuity of light perception. Optical penetrating keratoplasty was recommended; however, the patient declined any further surgical intervention.

**Fig. 4:**
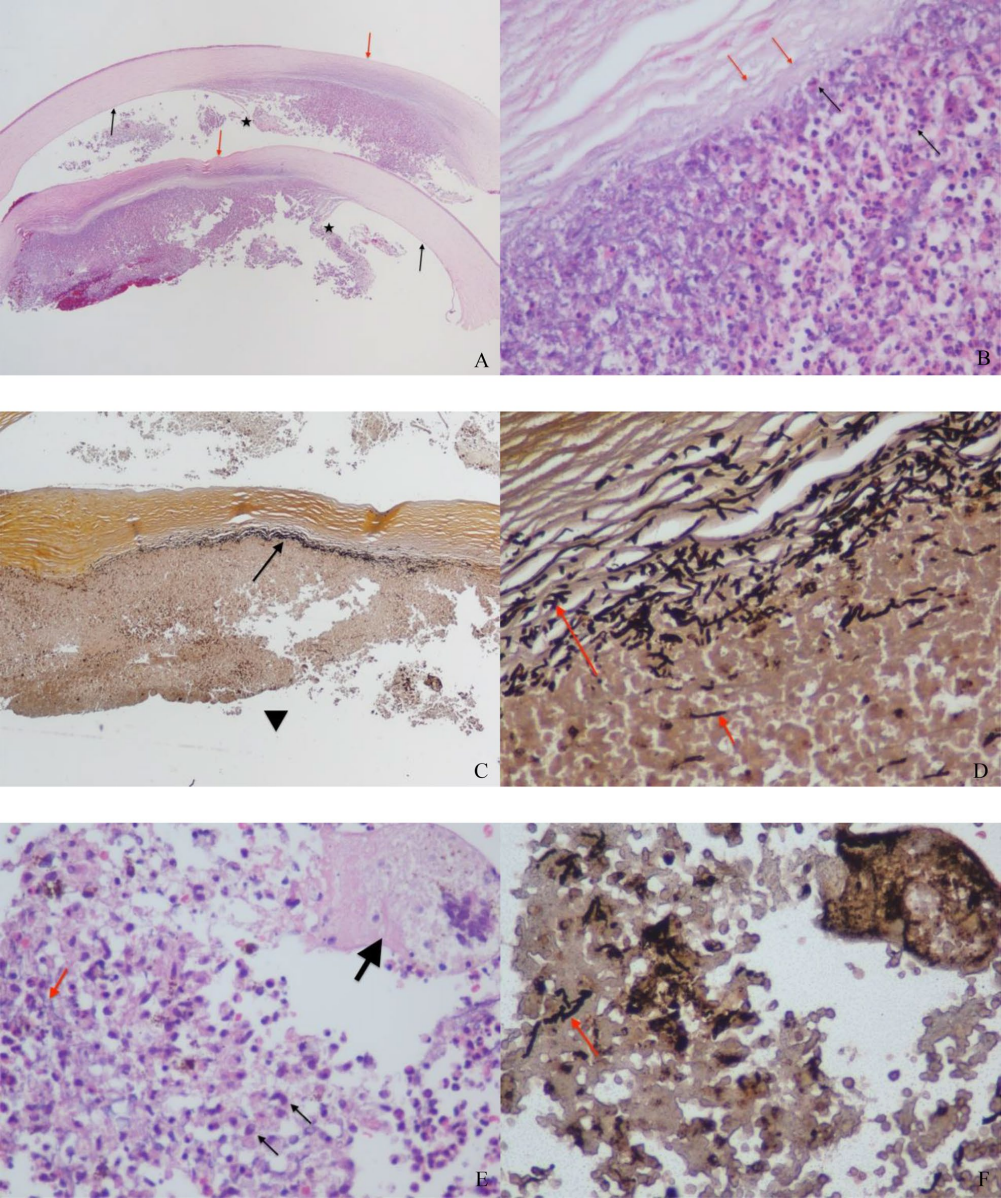
Histopathologic findings. **A (HE**,** ×20)**: A deep stromal abscess was observed anterior to Descemet’s membrane, which showed rupture with partial loss. (red arrow: intact Bowman’s layer; black arrow: Descemet’s membrane; pentagram: ruptured end of Descemet’s membrane). **B** **(HE, ×400)**: A high-magnification view highlighted abundant neutrophils within the abscess (black arrow) and faintly visible, pale basophilic fungal hyphae in cross-section in the deep stroma (red arrow). **C (GMS, ×40):** Numerous fungal hyphae (arrow) were visible within the deep stroma, located anterior to the abscess. The black triangle labels the anterior chamber side. **D (GMS**,** ×400)**: A high-magnification view highlighted dense hyphae (long red arrow) in the deep stromal layer and sparse hyphae (short red arrow) within the abscess. **E (HE**,** ×400) & F (GMS**,** ×400)**: A granulomatous nodule was evident, composed of multinucleated giant cells (thick black arrow, **E**) and numerous histiocytes (thin black arrow, **E**). These cells surround pale basophilic fungal hyphae (red arrow, **E**), which are clearly delineated in the corresponding GMS stain (red arrow, **F**)

We confirmed fungal keratitis through histopathology. Histopathological staining [hematoxylin and eosin (HE) and Gomori methenamine silver (GMS)] showed involvement of the middle to posterior stroma, Descemet’s membrane, and endothelium, with an intact Bowman’s layer. In HE staining, an abscess with extensive inflammatory infiltrate was observed in the deep stromal layer, located anterior to Descemet’s membrane. Rupture of Descemet’s membrane was observed, with partial loss. GMS staining revealed that quite intensely stained fungal hyphae predominantly aligned in the deep stromal layer anterior to the abscess, with sparse hyphae within the abscess. Granulomatous nodules formed by multinucleated giant cells and numerous histiocytes were identified surrounding the fungal hyphae, consistent with chronic granulomatous inflammation (Fig. 4). No fungal elements were apparent at the margins of the removed corneal tissue. Finally, microbiological culture was negative after 2 weeks of observation.

## Discussion

This is the first reported case of human *D. aspalathi* infection presenting with fungal keratitis, as well as the first discovery of *D. aspalathi* infection in China.

*Diaporthe* is a genus of Ascomycetous fungi, which has often been reported as plant pathogens, nonpathogenic endophytes, and saprobes [2]. Its asexual state is *Phomopsis*, a form genus of imperfect fungi in the Coelomycetous class and Sphaeropsidaceae order [3]. Since the name *Diaporthe* (1870) predates *Phomopsis* (1905), *Diaporthe* has been adopted as the generic name in most of the recent studies on this fungal group. *Diaporthe* spp., which can inhabit seeds, twigs, branches, and leaves of various host plants, are ubiquitous throughout the environment in soil, dust, and water [2, 3]. Pathogenic *Diaporthe* spp. are responsible for diseases on various plant hosts; some of them, such as soybean, are economically important worldwide [4]. However, these fungi are rarely pathogenic in humans or animals [5]. Since 1999, 13 *Diaporthe* spp. have been reported in association with human infections in 15 cases [6–17] (Table 1): unidentified *Phomopsis* (*n* = 3), *Diaporthe phaseolorum* (*n* = 3), *Phomopsis longicolla* (*n* = 1), *Phomopsis bougainvilleicola* (*n* = 1), *Diaporthe raonikayaporum* (*n* = 1), *Diaporthe sojae* (*n* = 1), *Diaporthe eres* or *Diaporthe nobilis* (*n* = 1), *Diaporthe miriciae* (*n* = 1), *Phomopsis phoenicicola* (*n* = 1), *Diaporthe oculi* (*n* = 1), and *Diaporthe pseudooculi* (*n* = 1). In the current case, we identified the causative fungus as *D. aspalathi* through mNGS. *D. aspalathi* is a major pathogen of soybean—first described in the southern United States in 1973—which causes southern stem canker and results in severe yield loss [18]. It is also the main causative agent of canker and dieback of rooibos (*Aspalathus linearis*) [19]. It was formerly known as *D. phaseolorum* var. *meridionalis* [20] and finally named *D. aspalathi* in 2006 by van Rensburg [19]. *D. aspalathi* is in the list of Chinese quarantine pests [21] and has not been reported to cause any plant diseases in China so far. However, it has been detected and intercepted several times in imported soybeans from the United States and Argentina at Chinese ports, such as Ningbo and Shenzhen. Nevertheless, to our knowledge, human *D. aspalathi* infection has never been reported previously.

**Table 1:** Diaporthe and phomopsis infections: clinical cases

Country, Year (Reference)	Fungi	Age/Sex of Patient	Activity Linked to Plants	Underlying Diseases	Concurrent Medication	Body Site	Lesion Type	Indolent Progress Without Systemic Inflammation	Surgery	Antifungals	Medication Time	In Vitro Antifungal Susceptibility Testing (MICs)	Result
USA, 1999	Unidentified *Phomopsis (Asexual)*	61/F	Gardener	Rheumatoid arthritis, Diabetes	prednisone, methotrexate	Finger	Subcutaneous abscess with osteomyelitis	4 months	Surgical drainage	Itraconazole	6 M	amphotericin B 0.25 µg/ml, fluconazole 2 µg/ml, itraconazole 0.125 µg/ml.	Cured
French Guiana, 2011	*Diaporthe phaseolorum*	60/M	Farmer (walked barefoot)	Positive HTLV-1 serology		Forefoot	Subcutaneous tumor with osteomyelitis	4 years		Itraconazole	> 6 M		Clinical remission
Spain, 2011	*Phomopsis longicolla (Asexual)*	72/F	Farmer (5 years ago)	Renal transplant	tacrolimus, mycophenolate, basiliximab, steroids	Finger	Cutaneous infection	1 year after transplantation	Surgical resection	Voriconazole	8 M	amphotericin B 0.03 mg/L, itraconazole 0.25 mg/L, voriconazole 0.015 mg/L, posaconazole 0.015 mg/L, terbinafine 1 mg/L, caspofungin < 0.03 mg/L.	Cured
Brazil, 2013	*Diaporthe phaseolorum (Asexual)*	43/M	Farmer	Renal transplant, Diabetes	antithymocyte globulin, tacrolimus and prednisone	Extremities	Cutaneous infection	2 months	Surgical resection	Itraconazole	5 M		Cured
USA, 2013	*Phomopsis boµgainvilleicola (Asexual)*	61/M	Carpenter	Renal transplant, Diabetes	tacrolimus, mycophenolate, prednisone	Knee	Bursitis (Subcutaneous tissue)	No systemic inflammation	Surgical drainage	Voriconazole	4 M	amphotericin B 1 µg/ml, anidulafungin 0.12 µg/ml, caspofungin 0.12 µg/ml, itraconazole 0.5 µg/ml, micafungin < 0.6 µg/ml, posaconazole < 0.06 µg/ml, voriconazole 0.5 µg/ml.	Cured
France, 2016	*Diaporthe sp (Asexual)*	78/M		Diabetes		Foot	Subcutaneous infection			Itraconazole	6 M		Relapse 6 M after ITRA
	*Diaporthe raonikayaporum (Asexual)*	51/M	Trauma	Renal transplant, Diabetes	Tac, mycophenolate mofetil, prednisone	Finger and foot	Subcutaneous infection		Surgical resection			amphotericin B 1 µg/ml, itraconazole 2 µg/ml, voriconazole 0.06 µg/ml, posaconazole 1 µg/ml, caspofungin 0.125 µg/ml, micafungin 0.125 µg/ml, terbinafin 4 µg/ml.	Cured
	*Diaporthe sojae (Asexual)*	68/M		B cell lymphoma	high-dose chemotherapy and long-term corticotherapy	Heel	Cutaneous infection			Voriconazole	10 W	amphotericin B 1 µg/ml, itraconazole 0.5 µg/ml, voriconazole 0.03 µg/ml, posaconazole 1 µg/ml, caspofungin 0.03 µg/ml, micafungin 0.25 µg/ml, terbinafin 1 µg/ml.	Regression, died
USA, 2017	*Diaporthe eres or nobilis*	79/M	Gardener	Heart transplant	tacrolimus, azathioprine, prednisone	Thigh	Subcutaneous soft tissue infection	6 months	Surgical resection	Posaconazole	3 M	posaconazole 0.25 µg/mL, voriconazole 0.25 µg/mL, caspofungin 0.25 µg/mL, amphotericin B 0.125 µg/mL, terbinafine 0.125 µg/mL.	Cured
New Zealand, 2019	*Diaporthe phaseolorum*	46/M	(a minor abrasion)	Heart transplant, end-stage renal failure	mycophenolate, tacrolimus; prednisone	Leg	Subcutaneous soft tissue infection	1 year	Surgical resection	Itraconazole	7 M		
Taiwan, 2022	*Diaporthe miriciae (Asexual)*	59/F	Farmer (repeated plant thorn injuries) (residue)	Diabetes		Hands and fingers	Cutaneous infection	1 month	Surgical resection	Terbinafine	2 M		Cured
USA, 2009	*Unidentified Phomopsis (Asexual)*	63/M	Gardener (rose thorn injury) (residue)		antibiotics, antivirus, topical steroids	Cornea	Keratitis	2 months (quiet)	PKP	Oral voriconazole, topical voriconazole, topical amphotericin B	5 M	sensitivity to both voriconazole and amphotericin B.	Cured
India, 2011	*Phomopsis phoenicicola (Asexual)*	48/M				Cornea	Scleral keratitis (Immediately after pterygium surgery)			Oral fluconazole, topical fluconazole, topical natamycin	2 M	natamycin > 32 µg/ml, fluconazole > 256 µg/ml, itraconazole > 256 µg/ml.	Cured
Japan, 2019	*Diaporthe oculi (Asexual)*	80/M	Farmer		antibiotics, topical steroids	Cornea	Keratitis	No systemic inflammation	PKP	Intravenous voriconazole, topical voriconazole	18 M	amphotericin B 0.25 µg/ml, flucytosine > 64 µg/ml, fluconazole 64 µg/ml, itraconazole 1.0 µg/ml, miconazole 2.0 µg/ml, micafungin 0.03 µg/ml, voriconazole < 0.015 µg/ml, pimaricin 2.0 µg/ml.	Cured
Japan, 2019	*Diaporthe pseudooculi (Asexual)*	68/M	Gardener (rose thorn injury) (residue)			Cornea	Keratitis	No systemic inflammation		Antibiotics first, Intravenous voriconazole, topical voriconazole, topical amphotericin B, topical pimaricin	10 M	amphotericin B 0.25 µg/ml, flucytosine > 64 µg/ml, fluconazole > 64 µg/ml, itraconazole 1.0 µg/ml, miconazole 2.0 µg/ml, micafungin 0.06 µg/ml, voriconazole 0.12 µg/ml, pimaricin 4.0 µg/ml.	Cured

Note: MICs: Minimum Inhibitory Concentrations; PKP: Penetrating Keratoplasty

Table 1 provides an overview of 15 cases of human *Diaporthe* infection reported so far. Most (80%) of the patients were male (sex ratio = 4:1), and the median age was 62.5 years (range, 43–80 years). Moreover, 10 (67%) patients were farmers, gardeners, or carpenters. Five patients had a history of trauma, with three having a residual thorn. Underlying diseases were reported in 11 (73%) cases. Six patients had received solid organ transplantation (kidney, *n* = 4; heart, *n* = 2), six had diabetes mellitus, one had B-cell lymphoma, one had rheumatoid arthritis, and one was positive for human T-lymphotropic virus 1 in serologic testing. Topical or oral steroids and immunosuppressive agents were administered to 10 patients. Cutaneous or subcutaneous infections (such as nodules, abscesses, infiltrated plaques, osteomyelitis, or bursitis) in distal body areas were noted in 11 patients, whereas keratitis was observed in 4 patients.

Trauma or contact with a contaminated source (e.g., exposure of a body part to a plant or soil), residual foreign body, underlying immunodepression, or diabetes mellitus are potential risk factors for an opportunistic infection. The incubation period before localized infection development without systemic manifestations, such as fever or abnormal laboratory test results, may be prolonged (months to years). Thus, although organic matter may not have sufficient pathogenicity and virulence, it may persist in a subclinical or latent form and subsequent immunosuppression may trigger an active infection. In 12 (80%) of the aforementioned cases, fungi were identified in their asexual state, *Phomopsis*, based on their morphological characteristics in culture. This state is most common, often observed in many major diseases [16]. Because of the rarity of *Diaporthe* infections, an optimal treatment protocol has not been defined thus far. In the 15 aforementioned patients, 5 were treated with antifungal agents alone, 1 was treated via excision alone, and 9 were treated through a combination of surgery and antifungal agents. Of the five patients treated with antifungal agents alone, one died of the underlying disease within the first 3 months, one relapsed, and one achieved clinical remission. In the remaining 12 (80%) cases, complete resolution was achieved. In vitro antifungal susceptibility testing was performed in 10 cases, only to determine reference values because an optimal *Diaporthe* susceptibility testing method has not been established. Of all 15 cases, 9 demonstrated efficacious responses to the oral triazole agents (itraconazole, voriconazole, and posaconazole).

*Diaporthe* fungi previously reported to cause keratitis include an unidentified *Phomopsis*, *P. phoenicicola*, *D. oculi*, and *D. pseudooculi.* In the current case, we identified *D. aspalathi* as the fifth novel keratitis-causing *Diaporthe* spp. in humans. However, its teleomorph or anamorph could not be distinguished because the culture results were negative. Compared with the cases caused by the four aforementioned *Diaporthe* spp., the present case posed greater diagnostic and therapeutic challenges.

This case underscored critical diagnostic challenges in fungal keratitis. Despite a clear history of thorn injury, the patient received a prolonged course of antibiotic eye drops, followed by a misdiagnosis of HSK and inappropriate steroid use that exacerbated the infection. Histopathologic findings indicated that the lesion was deeply located, likely due to direct fungal inoculation into the deep stroma by the thorn, resulting in abscess formation. This deep location rendered both confocal microscopy and corneal scrapings non-diagnostic. Collectively, these factors contributed to a significant diagnostic delay. Similar errors have been reported in previous cases of *Diaporthe* keratitis following thorn trauma, including initial antibiotic use [7, 16] and misdiagnosis as HSK [7]. Therefore, maintaining a high index of suspicion for fungal keratitis following any plant-related corneal injury, even those with a prolonged latency, is crucial. In cases where deep corneal involvement is suspected, mNGS provides a rapid and reliable diagnostic approach, requiring only a minimal clinical sample.

The treatment course in this case was also challenging. Despite an aggressive therapeutic approach including penetrating keratoplasty, the clinical outcome was unfavorable, characterized by graft opacity, rejection, and suspected fungal recurrence, necessitating 3 months of topical and systemic antifungal therapy. Several factors contributed to this complexity: (1) Retained Foreign Body: A residual thorn, previously reported in similar injuries [7, 16], likely acted as a persistent fungal nidus, highlighting the imperative for meticulous examination and removal of corneal foreign bodies. (2) Deep Stromal Involvement: Histopathology confirmed that the infection was localized predominantly in the deep stroma, with abscess formation leading to Descemet’s membrane penetration. This deep-seated nature likely limited topical drug efficacy, underscoring the need for early surgical intervention in such cases. (3) Antifungal Strategy: The lack of drug susceptibility data and poor corneal penetration of topical agents complicated treatment. A postoperative regimen combining topical 5% natamycin and 2% voriconazole with systemic voriconazole proved effective. This aligns with previous reports of *Diaporthe* keratitis, suggesting that a combination of polyenes and triazoles, administered both topically and systemically, constitutes a crucial therapeutic approach. Based on this efficacy, oral voriconazole warrants consideration as a first-line agent for *Diaporthe* keratitis, though further studies are needed to optimize antifungal regimens. (4) Risk of Recurrence: Two factors may have contributed to recurrence risk. First, although a sufficiently large corneal graft was employed, its inclusion of inferior corneoscleral tissue—the presumed site of the residual thorn—may have increased recurrence risk. Infections involving the limbus have recurrence rates as high as 20.69% [22]. Second, the presence of sparse hyphae within the abscess and their potential dissemination into the anterior chamber following Descemet’s membrane rupture may have allowed fungal persistence, despite intensive intraoperative irrigation.

## Conclusion

*D. aspalathi*, a plant pathogen, should be added to the list of human pathogenic fungi. It is a rare causative agent of fungal keratitis in humans and is difficult to diagnose and treat. Therefore, in patients with corneal damage due to a plant part, fungal keratitis should be strongly suspected. Clinicians should thoroughly examine patients for residual foreign bodies. Topical steroids should be avoided. In severe cases, a combination of antifungal medications and surgical intervention is recommended.

Moreover, although *D. aspalathi* has not been officially reported to cause plant diseases in China, we suspect that this pathogen may already be present in local flora. To mitigate potential risks, government agricultural agencies should develop and implement containment strategies to prevent *D. aspalathi* from spreading to disease-free soybean-growing regions across the country.

## Data Availability

All data generated or analyzed during this study are included in this published article.

## Abbreviations

HSK: Herpes simplex keratitis
mNGS: Metagenomic next-generation sequencing
HE: Hematoxylin and eosin
GMS: Gomori methenamine silver
